# Pathogenic Pore Forming Proteins of *Plasmodium* Triggers the Necrosis of Endothelial Cells Attributed to Malaria Severity

**DOI:** 10.3390/toxins13010062

**Published:** 2021-01-15

**Authors:** Abhishek Shivappagowdar, Swati Garg, Akriti Srivastava, Rahul S. Hada, Inderjeet Kalia, Agam P. Singh, Lalit C. Garg, Soumya Pati, Shailja Singh

**Affiliations:** 1 Department of Life Science, School of Natural Sciences, Shiv Nadar University, Chithera, Gautam Buddha Nagar, Uttar Pradesh 201314, India; as775@snu.edu.in (A.S.); as598@snu.edu.in (A.S.); rh144@snu.edu.in (R.S.H.); soumya.pati@snu.edu.in (S.P.); 2 Special Centre for Molecular Medicine, Jawaharlal Nehru University, New Delhi 110067, India; swati.garg@snu.edu.in; 3 Infectious Disease Lab, National Institute of Immunology, New Delhi 110067, India; inderjeet@nii.ac.in (I.K.); singhap@nii.res.in (A.P.S.); 4 Gene Regulation Laboratory, National Institute of Immunology, New Delhi 110067, India; lalit@nii.ac.in

**Keywords:** malaria, *Plasmodium falciparum*, perforin like proteins, necrosis, blebbing, calcium, HMGB1

## Abstract

Severe malaria caused by *Plasmodium falciparum* poses a major global health problem with high morbidity and mortality. *P. falciparum* harbors a family of pore-forming proteins (PFPs), known as perforin like proteins (PLPs), which are structurally equivalent to prokaryotic PFPs. These PLPs are secreted from the parasites and, they contribute to disease pathogenesis by interacting with host cells. The severe malaria pathogenesis is associated with the dysfunction of various barrier cells, including endothelial cells (EC). Several factors, including PLPs secreted by parasites, contribute to the host cell dysfunction. Herein, we have tested the hypothesis that PLPs mediate dysfunction of barrier cells and might have a role in disease pathogenesis. We analyzed various dysfunctions in barrier cells following rPLP2 exposure and demonstrate that it causes an increase in intracellular Ca^2+^ levels. Additionally, rPLP2 exposed barrier cells displayed features of cell death, including Annexin/PI positivity, depolarized the mitochondrial membrane potential, and ROS generation. We have further performed the time-lapse video microscopy of barrier cells and found that the treatment of rPLP2 triggers their membrane blebbing. The cytoplasmic localization of HMGB1, a marker of necrosis, further confirmed the necrotic type of cell death. This study highlights the role of parasite factor PLP in endothelial dysfunction and provides a rationale for the design of adjunct therapies against severe malaria.

## 1. Introduction

Malaria is probably one of the oldest life-threatening diseases caused by the protozoan parasites of the genus *Plasmodium*. The mortality due to malaria is extremely high, with children below the age of five to be most susceptible, and this contributes to 67% of death caused by malaria globally [1]. While uncomplicated *Plasmodium falciparum* infection is highly treatable, the most severe complication involves the central nervous system (cerebral malaria (CM)) that sustains neurological impairment and is potentially fatal. Human cerebral malaria (HCM) is characterized by disruption of the blood–brain barrier (BBB), coma, seizures, and death. Patients that survive HCM are prone to developing cognitive and neurological deficits after recovery from the infection [2]. Although antimalarial drugs have shown efficacy in killing parasites, treatments to improve the outcome of severe malaria are lacking due to the poor understanding of the cellular mechanisms regulating disease progression [3]. The risk factor of brain injury associated with studying human patients during infection limits our understanding of severe malaria and has created a need for additional preclinical research.

The pathophysiology of severe malaria is extremely complex, and is a field of intense research. The three main mechanisms are sequestration of infected RBCs, which adhere to the vascular endothelium and produce a cascade of intracellular signaling in these cells, thus inducing the activation of endothelial cells; triggering of immunological and systemic inflammatory responses; and coagulation dysfunction [4,5,6,7]. The dynamic interaction among all the three mechanisms accounts for the pathogenesis of HCM, explaining the complexity of this potentially fatal infection [8]. Parasitized RBCs (pRBCs) express several parasite-encoded molecules on their surfaces, notably *Plasmodium falciparum* erythrocyte membrane protein-1 (PfEMP-1), which protrudes through the pRBC surface and interacts with several host receptors, such as CD36, ICAM-1 (cerebral malaria), and CS-A (placental malaria) [9,10]. This binding causes the cytoadhesion of pRBCs to the vascular endothelial cells, which further form rosettes with other non-pRBCs and sequester with host leukocytes and platelets that finally slow down the blood circulation due to capillary hindrance. This results in a hypoxic condition that worsens the coma. However, in the case of an adhesion independent pathway, ligand receptor specific interactions are not required. These pathways activate a secondary signaling cascade, which includes the induction of a toxic local microenvironment, metabolic competition, and the release of parasite products, which act as neurotoxins and other neuroactive mediators [11]. All of these factors cause cellular damage, which could influence the expression of junctional proteins and damage cells including microglia, astrocytes, neurons, and other parenchymal cells [12].

Previous studies have stated that the adhesion of pRBCs to the endothelial cells (ECs) induce cell apoptosis via nitric oxide and oxidative stress [13]. Moreover, in vivo studies suggest that the cytotoxic effector CD8+ T lymphocytes are implicated in the pathogenesis of murine CM perforin-dependent cytotoxicity against the ECs [14]. A recent study has demonstrated that the trypsin-resistant membrane components of *Plasmodium falciparum*-infected red blood cells (Pf-IRBCs) and parasite-secreted soluble factors contribute to the disruption of BBB integrity. This result was established by measuring the decrease in human brain microvascular endothelial cells (HBMECs) electrical resistance [5]. However, the exact soluble factors causing the disturbance in the BBB were not identified. The experimental cerebral malaria (ECM) model utilizes *Plasmodium berghei* ANKA (PbA) infection to several mouse strains, mainly C57/BL6, for determining mechanistic details that help to introduce rationale to be tested in humans. The ECM system shares many features similar to HCM. Precisely, in both ECM and HCM, parasitic infection initiates alteration in BBB and/or a locally restricted rupture of BBB leading to hemorrhages. During ECM, the mechanism of disease commencement may involve the accumulation of pRBCs, leukocytes sequestration, and an influx of CD8+ T cells, macrophages, and neutrophils into the brain [13,15]. In addition to immune infiltration, low nitric oxide bioavailability appears to play a role in ECM pathogenesis [16].

*Plasmodium falciparum* pore-forming proteins (PFPs), known as perforin like proteins (PLPs), are the critical drivers of the parasite life cycle [17,18]. They have a role in sporozoite infection, merozoite egress, gametocyte egress, and transversal of mosquito midgut to form ookinetes [19,20,21,22,23,24,25]. They share structural similarity with bacterial PFPs, both containing a central pore-forming domain comprising a bent and twisted β-sheet that is flanked by three clusters, usually comprising α-helices [26]. Out of the five *Plasmodium falciparum* perforin like proteins (PPLPs), PPLP2 is known to be expressed during the blood stage as well as in mature gametocytes [19,21]. PPLP2 has a critical role in the egress of activated gametocytes through the permeabilization and rupture of the RBC membrane [19]. In addition, *P. berghei* PLP2 knockout mutants signify that PLP2 has a critical role in the egress of male gametocytes from infected RBCs, contrary to female gametocytes, which developed and egressed normally [20]. PPLP2 has a central MACPF domain and an N-terminal signal sequence [21].

Studies have shown that the human perforin secreted by CD8+ T cells causes endothelial disruption and fatal edema [16]. In our previous work [27], we have demonstrated that the highly conserved region of *Plasmodium* PLPs is a membrane attack complex/perforin (MACPF) domain that can form pores on human RBCs leading to its lysis. Moreover, PLP induced an increase in intracellular calcium, leading to premature RBC senescence. Since it is known that the soluble factors of *P. falciparum* play an important role in the pathogenesis of CM, we hypothesized that the release of PLPs at the cerebral vasculature can lead to severe neurological dysfunction. Hence, we designed a model system using epithelial as well as endothelial cells to study the pathogenesis of PLPs in the context of severe malaria.

Here, we have investigated whether PPLP2’s membrane permeabilization activity has any role in the rupture of EC and BBB. We have therefore expressed the PPLP2 MACPF domain in *E. coli* and purified the recombinant PLP2 (rPLP2). ECs were treated with rPLP2 to further deduce the effect with respect to severe malaria. Our data indicate that rPLP2 induces an increase in intracellular Ca^2+^ levels in primary vascular cells, HUVEC, and MDCK cells. Additionally, rPLP2 exposed ECs also exhibit features of apoptotic cell death, characterized by the exposure of phosphatidylserine and propidium iodide positivity, loss of mitochondrial membrane potential, increased reactive oxygen species (ROS) generation, and high mobility group box 1 (HMGB1) release. In conclusion, this study indicates the role of PLPs in ECs, thus providing new insights into severe malaria pathogenesis.

## 2. Results

### 2.1. rPLP2 Induces Death of Primary Endothelial Cells In Vitro

**Figure 1:**
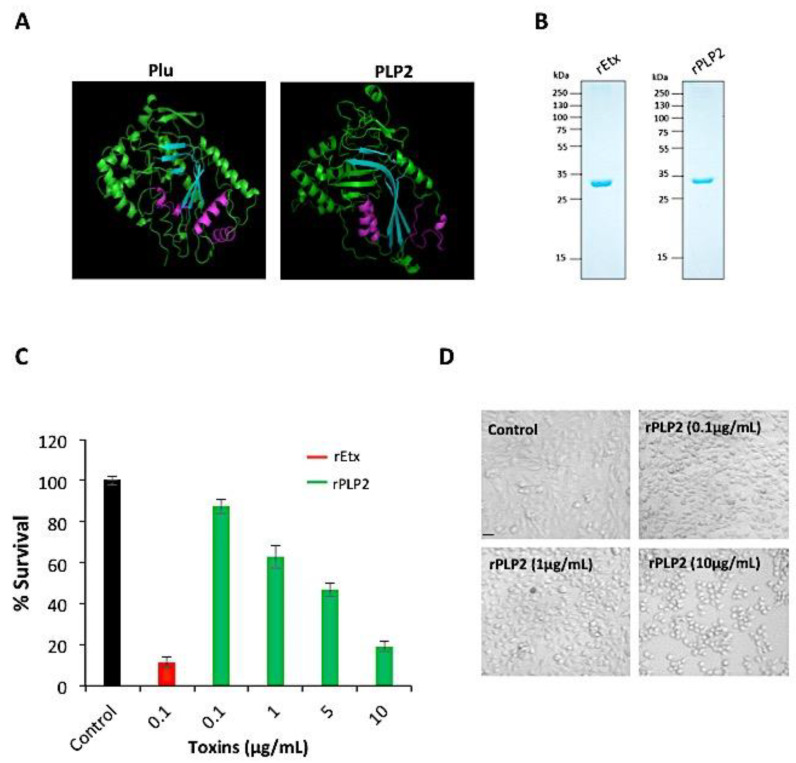
Activity of rPLP2. (**A**) The tertiary structure conservation analysis between the MACPF domains of *Photorhabdus luminescens* Plu (2qp2) and *P. falciparum* PLP2 (predicted model) show a high degree of similarity between them. The conserved regions are shown in the same color. The central β-sheet is represented in cyan, and the transmembrane hairpins (TMH) on either side are shown in purple. (**B**) The Coomassie-stained SDS-PAGE gel image of rPLP2 and rEtx is shown along with molecular weight markers. (**C**) MDCK cells were challenged with rEtx (0.1 µg/mL (3nM)) or the different concentrations of rPLP2 (0.1 µg/mL (3 nM), 1 µg/mL (31 nM), 5 µg/mL (157 nM) and 10 µg/mL (313 nM)), and the survival was affirmed by an MTT assay. The error bar represents the mean ± SD. (**D**) The microscopic analysis of the effect of rPLP2 on MDCK cells was monitored by incubating the cells with different concentrations of the protein. Control cells were incubated with media only. The scale bar represents 20 μm.

Structural study of Plu-MACPF, a bacterial protein from *Photorhabdus luminescens* [28], revealed that all MACPF domain has a conserved signature motif of (Y/W)-X6-(F/Y)GTH(F/Y)-X6-GG and two transmembrane helices (CH1 and CH2), irrespective of their prokaryotic or eukaryotic origin. Also, both eukaryotic and bacterial MACPF domains have structural similarity and a similar membrane insertion mechanism. Towards this, we performed in silico analysis to compare the PLP2-MACPF structure to the Plu-MACPF X-ray crystal structure (2qp2) [28]. Our data indicated significant conservation of the MACPF domain between Plu-MACPF and PLP2, with two transmembrane helices present on either side of the β-sheet (Figure 1A).

**Figure 2:**
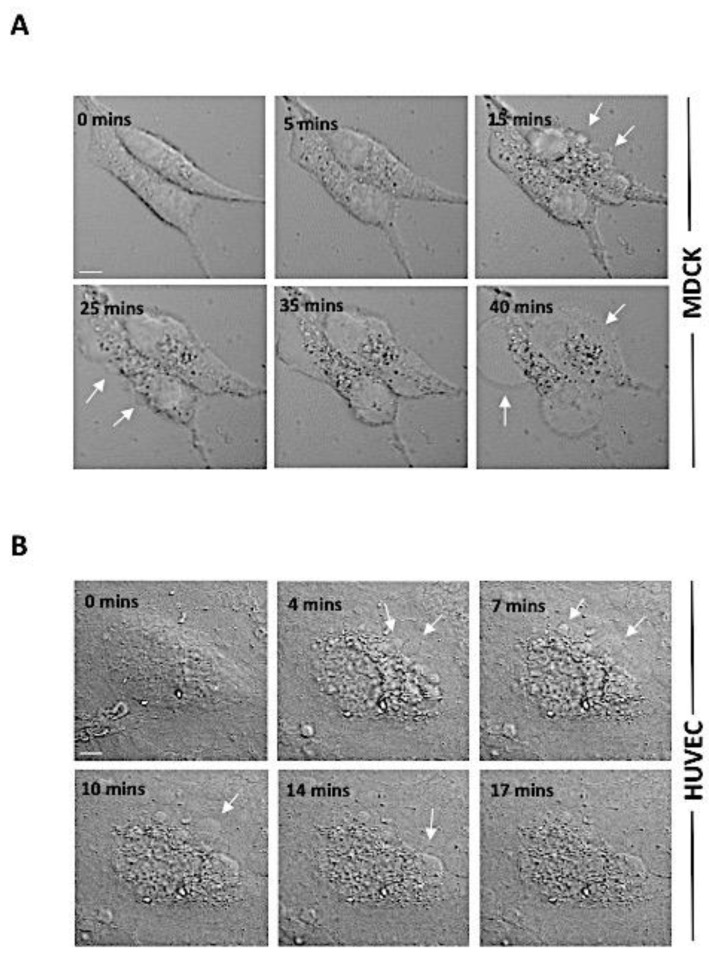
Membrane blebbing of the cells in response to rPLP2. The MDCK cells (**A**) and HUVEC (**B**) were treated with rPLP2 (10 µg/mL), and the changes in the membrane architecture were observed in real time by confocal microscopy. Selected images of DIC with time elapsed between frames in minutes (mins) are shown. Arrows indicate the blebbing of the cell membrane. The scale bar represents 2 μm.

Since both the eukaryotic and bacterial PFPs share a common method of pore formation, we studied and compared the activity of PLP2 with respect to epsilon toxin (a PFP secreted by *C. perfringens*). Initially, we purified both of the proteins in accordance with a previous report from our group [27,29]. A single band could be detected on the SDS-PAGE at their expected molecular weight, indicating the purity of the proteins (Figure 1B). The endotoxin level of the rPLP2 and rEtx was also estimated and was found to be 8.81 × 10^−6^ EU/mL and 8.26 × 10^−6^ EU/mL, respectively (Supplementary Figure S1A). To test the lytic activity, rPLP2 was incubated with RBCs and the hemoglobin release was monitored. rPLP2 demonstrated potential lytic activity against RBCs. The denatured rPLP2 was used as a negative control (Supplementary Figure S1B). To test the cytotoxic activity of rPLP2 on MDCK cells, we performed an in vitro MTT assay. This is a colorimetric assay for measuring the metabolic activity of the cell as an indicator of its viability or proliferation. It is based on the reduction of the MTT by the mitochondrial enzyme lactate dehydrogenase to insoluble formazan crystals, which exhibit a purple color upon the addition of an appropriate solvent. Briefly, rPLP2 was incubated with MDCK cells at different concentrations for 1 h at 37 °C. The viability of the cells decreased in a dose-dependent manner with an increase in rPLP2 concentration. The lethal effect of rPLP2 on MDCK cells was observed, starting from 100 ng/mL, and the IC_50_ was achieved at a concentration of 5 µg/mL. The data were normalized to values obtained for cells untreated with the protein (Figure 1C). A similar PFP of *Clostridium* spp, epsilon toxin (Etx), was used as a positive control. Furthermore, to decipher if the lethality caused by rPLP2 correlates with the morphological changes in MDCK cells, we performed light microscopy of rPLP2 treated cells. Similar to other toxins [30,31], we noticed that the rPLP2 treated cells displayed changes in the cellular architecture, in particular membrane blebbing and vesiculation, in a dose-dependent manner (Figure 1D). Next, we investigated whether the primary cell line HUVEC shows similar morphological changes due to rPLP2 toxic action. As shown (Figure 2A,B, Supplementary Figure S2A,B, and Supplementary Videos S1 and S2), the rPLP2 effect on the cells could be detected within a few minutes of treatment. Most of the cells developed rounded structures called blebs that increased and grew over time. These blebs are quasi-spherical protrusions typically formed on the cell membrane due to harsh membrane injury [32].

### 2.2. rPLP2 Mediated an Increase in the Intracellular Calcium Levels of Barrier Cells

**Figure 3:**
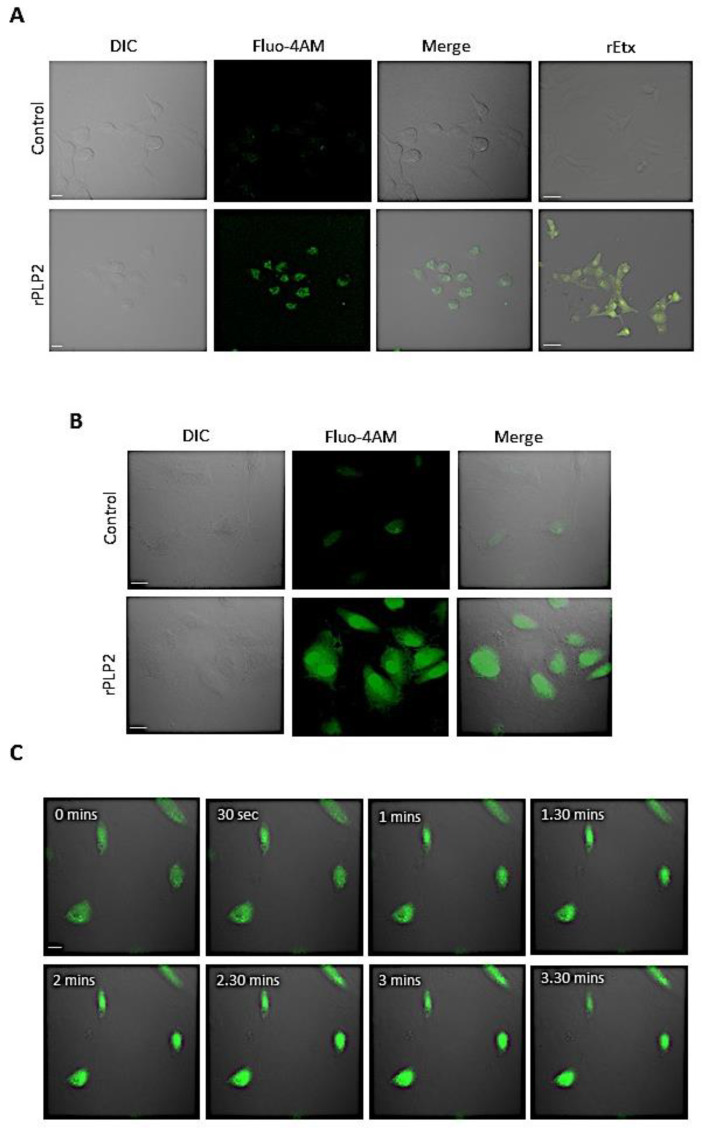
rPLP2 mediates calcium influx. Intracellular Ca^2+^ levels were monitored in MDCK (**A**) and HUVEC (**B**) cells loaded with Fluo-4 AM in the presence or absence of rPLP2 (5 µg/mL). (**C**) Time-lapse video microscopy was performed to monitor the increase in calcium levels in HUVEC after the addition of rPLP2 (5 µg/mL). Selected frames of fluorescent images merged with DIC images with time elapsed between frames are shown. The scale bar indicates 10 μm.

PFPs form pores in the plasma membrane through insertion that leads to ionic imbalance and causes the constant inflow of calcium, ultimately leading to cell death [33]. Hence, to check for the increase in the calcium levels, we stained the rPLP2 treated cells with Fluo-4 AM (a calcium sensitive dye). The rPLP2 treated MDCK and HUVEC cells displayed a clear increase in the intracellular Ca^2+^ levels compared to the control cells (Figure 3A,B). Calcium concentrations play a pivotal role in maintaining cellular homeostasis by balancing cell death and cell survival response against any stress stimuli [34]. To confirm that the calcium was entering through rPLP2 pores from extracellular media, we accessed the calcium influx in erythrocytes in the presence or absence of EGTA and Lanthanum Chloride (LaCl_3_), a Ca^2+^ chelator, and Ca^2+^ channel blocker, respectively. We demonstrated that calcium influx was highly reduced in EGTA and LaCl_3_ treated erythrocytes (Supplementary Figure S3A). To further confirm the involvement of extracellular calcium, we blocked the Ca^2+^ influx triggered by rEtx. To achieve this, we treated the cells with rEtx and observed the lethal effect in the presence or absence of EGTA and LaCl_3_. As expected, rEtx exposure led to a decrease in cell survival (Supplementary Figure S3B). However, upon treatment with EGTA (extracellular calcium chelator), an increase in survival was observed compared to rEtx alone. Similarly, when the calcium channels were blocked using Lanthanum Chloride (LaCl_3_), higher levels of cell viability were recorded.

Further, we tested whether the combination of EGTA and LaCl_3_ plays any significant role in MDCK survival. Our results demonstrated no synergetic effect on the protection of rEtx treated MDCK in the presence of both EGTA and LaCl_3_ (Supplementary Figure S3B). This indicated that extracellular calcium plays a major role in rEtx induced toxicity of MDCK cells. In concordance with the above finding, we hypothesized that the Ca^2+^ influx might also have a role in the case of rPLP2 treated cells. Therefore, to monitor more closely, we performed time-lapse microscopy of HUVEC cells incubated with rPLP2 and observed a sudden increase in intracellular calcium of HUVEC cells following the addition of rPLP2. The calcium influx might further lead to morphological changes in the cells, eventually leading to cell death (Figure 3C and Supplementary Video S3).

### 2.3. rPLP2 Leads to the Exposure of Phosphatidylserine and Propidium Iodide Positivity Resulting in Late Apoptosis/Necrosis

**Figure 4:**
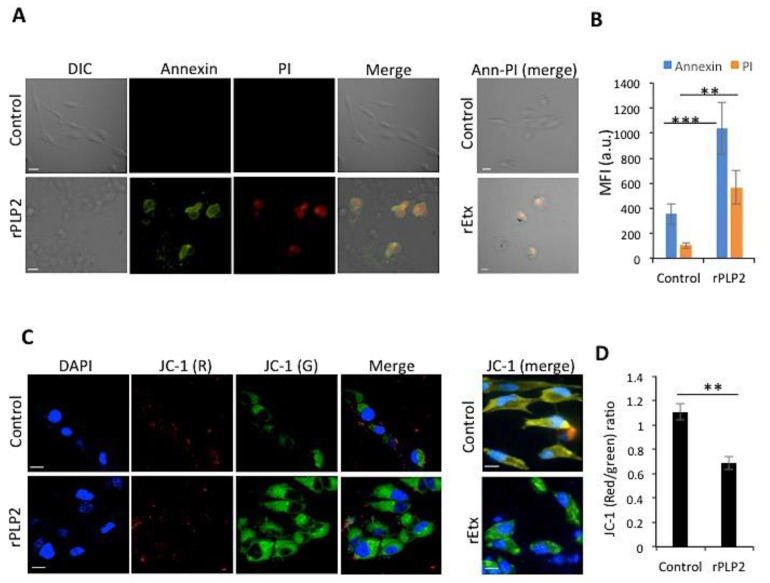
rPLP2 treated cells express markers of cell death. (**A**) MDCK cells were treated in the presence and absence of rPLP2 (5 µg/mL) and the Annexin V/PI staining was observed. The scale bar represents 10 μm. rEtx treated MDCK cells were taken as a control. (**B**) The bar graph depicts the changes in the mean fluorescence intensity (MFI) for both Annexin and PI between control and treated groups. (**C**) MDCK cells were loaded with JC-1 and the effect on mitochondrial membrane depolarization (ΨΔm) was observed. The scale bar represents 10 μm. rEtx (50 ng/mL) treated MDCK cells were taken as a control. (**D**) Bar graph depicts the red (aggregates)/green (monomers) ratio. Error bars represent mean ± SD. The MFI was quantified for a minimum of 40 cells for each treatment in the case of both (**B**) and (**D**). Statistical significance is shown for rPLP2 vs. control (** *p* < 0.01, and *** *p* < 0.005).

To analyze whether rPLP2-mediated cell death was due to a lytic or non-lytic mechanism (i.e., necrosis or apoptosis), cells incubated with rPLP2 were monitored for exposure of phosphatidylserine and uptake of propidium iodide. Cells that were positive for Annexin V alone signified apoptosis, as it indicated the exposure of phosphatidylserine without cell permeabilization. On the contrary, cells that were positive only for PI were considered necrotic, indicating permeabilization. However, Annexin V/PI double positive cells were considered late apoptotic/necrotic. Treatment of the cells with rPLP2 showed a clear Annexin V and PI dual positivity, confirming cell death (Figure 4A). Moreover, the mean intensity graph showed a clear difference between the control and treated cells (Figure 4B). This data indicated that rPLP2 causes late apoptotic/necrotic of MDCK cells. Epsilon toxin was used as a control, denoting its similar activity to rPLP2 (Figure 4A). The accumulation of calcium in the mitochondria contributes to the disruption of mitochondrial membrane potential (ΨΔm), causing lower levels of ATP production that proceed to cell death [35]. Hence, to monitor the effect of rPLP2 on the mitochondrial disruption of MDCK cells, we used JC-1 dye, which is an indicator of ΨΔm. In the case of healthy cells, the mitochondria showed a potential-dependent accumulation of JC-1, leading to the formation of J aggregates. However, in the case of depolarization, JC-1 existed in its monomeric form, leading to green fluorescence. The depolarization was indicated by a decrease in the red/green fluorescence intensity ratio. Upon treatment of the cells with rPLP2, a rapid decrease in the mitochondrial membrane potential was observed, as evident by the increase in the accumulation of green monomers (Figure 4C, Supplementary Figure S4). An unambiguous decrease in the red/green fluorescence intensity ratio could be observed as compared to the control cells, which had intact potential (Figure 4D). rEtx treated MDCK cells were used as a positive control.

### 2.4. rPLP2 Increases the Levels of Intracellular Reactive Oxygen Species in Primary Cells

**Figure 5:**
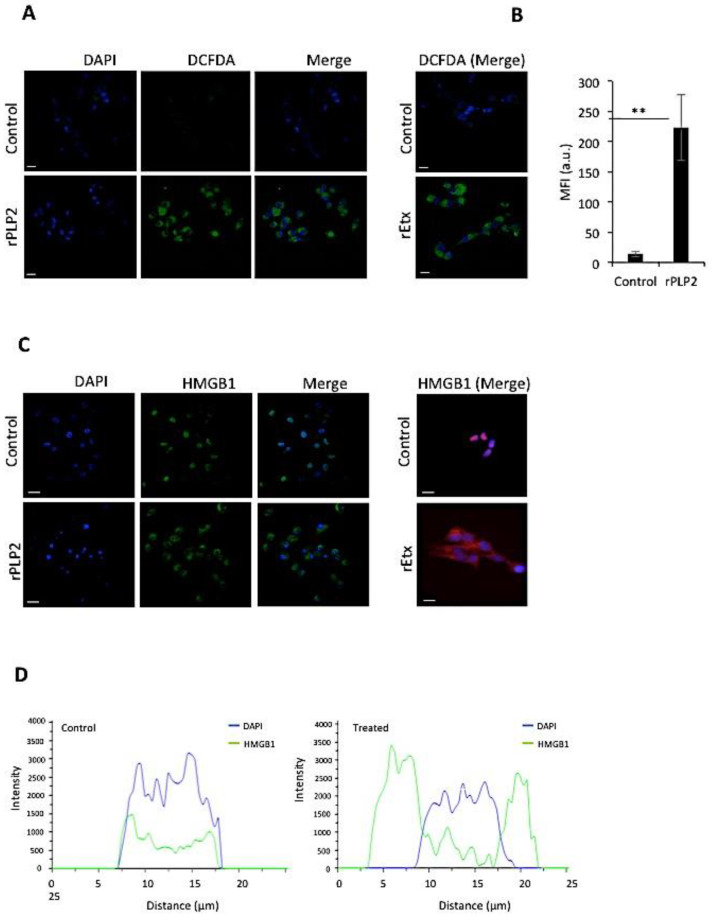
rPLP2 treated cells show ROS increase and HMGB1 translocation. (**A**) Intracellular DCFDA levels in the presence and absence of rPLP2 (5 µg/mL) were monitored in cells. The scale bar indicated 10 μm. rEtx (50 ng/mL) treated MDCK cells were taken as a control. (**B**) The bar graph represents MFI for DCFDA between the control and treated groups. The fluorescence was quantified for a minimum of 50 cells for each treatment. Error bars represent mean ± SD. ** *p* < 0.01 vs. control. (**C**) The localization of HMGB1 was monitored in the presence and absence of rPLP2. rEtx treated MDCK cells were taken as a positive control. (**D**) Co-localization line profiles of DAPI (blue) and HMGB1 (green) are represented. The scale bar represents 10 μm.

Excess production of ROS by the cells can cause damage to DNA, proteins, and lipids, leading to the activation of the cell death pathway [36]. rPLP2 could enhance intracellular ROS production that may result in cell death. Hence, we monitored the ROS production in cells using a cell-permeant DCFDA dye (2′,7′-dichlorodihydrofluorescein diacetate). This dye, in the presence of ROS, exhibits higher levels of fluorescence, thus providing a reliable detection method. For cells that were not treated with rPLP2, basal levels of ROS could be detected (Figure 5A). However, in rPLP2 treated MDCK cells, higher levels of ROS could be observed. A bar graph shows a clear shift in the fluorescence intensity between the control and treated cells (Figure 5B). A similar increase in ROS could be detected in the MDCK cells exposed to rEtx, as described earlier [37].

### 2.5. rPLP2 Treatment Leads to the Translocation of HMGB1 from the Nucleus to the Cytosol

In healthy cells, HMGB1 accumulates in the nucleus and is found at sites of oxidative DNA damage, thus functioning in DNA repair [38]. However, in the case of necrosis, it functions as a cytokine mediator of inflammation. Studies indicate that ROS can release HMGB1 through calcium/calmodulin-dependent kinase-mediated signaling [39]. Hence, to check for the localization of HMGB1, we performed an immunofluorescence assay in the presence or absence of rPLP2. In rPLP2 untreated cells, the HMGB1 was localized in the nucleus with complete absence in the cytoplasm. Conversely, upon treatment of MDCK cells with rPLP2, HMGB1 migrated from the nucleus to the cytosol, indicating cell necrosis (Figure 5C). Co-localization intensity plot analysis for DAPI and HMGB1 suggested that HMGB1 was present in the nucleus in untreated cells, while, in the case of treatment, co-localization was lacking, indicating the migration of HMGB1 from the nucleus to the cytoplasm (Figure 5D). Hence, both rEtx (positive control) and rPLP2 exhibited similar activity and led to the necrosis of their target cells in vitro.

## 3. Discussion

The major functions of the BBB are to mainly provide a controlled entry and exit of various molecules that infiltrate the brain through the EC tight junctions and its membrane proteins [40]. Through extensive studies in animal models, cultured cells, and patients, it has become evident that the dysfunction of barrier cells plays a critical role in the development of severe malaria. Several factors, including inflammatory cytokines and ROS, have been implied in its disruption [41]. It is usually seen that in the case of severe malaria, the Pf-IRBCs adhere to the EC through a series of sequential and specific interactions [8], which results in the obstruction of the vasculature, in turn blocking the normal blood supply and the movement of other molecules. Along with this, the toxins released by the Pf-IRBCs can have a major role in the disruption of the BBB. Altogether, the parasitic infection can cause cerebral edema, along with hemorrhages and occlusions, imposing deadly sequelae of neurological injury, promoting disease severity [42].

It is well known that *P. falciparum* secretes PLPs that play an important role in the progression of its life cycle [21,43]. Our previous study [27] demonstrated that PLPs form pores on the RBCs, leading to their lysis in the blood stage. Herein, we hypothesize that the rupture of Pf-IRBCs after schizogony leads to the release of PLPs, which in turn compromises endothelial barrier integrity, leading to the death of ECs.

To initially check the lethal effect of rPLP2 on mammalian cells, we performed an MTT assay. As shown in the Figure 1C, MDCK cells showed dose-dependent lysis, with an increase in rPLP2 treatment. Since the pore-forming toxins (PFTs) exposed cells often display changes in membrane architecture, we performed light microscopy. rPLP2 exposed cells showed changes in the cell morphology, including cell rounding and membrane shrinkage (Figure 1D). Further, to observe the membrane changes in real time, we performed live-cell video microscopy. The rPLP2 treated cells initially displayed swelling at different sites on the membrane called blebs (Figure 2A,B). Moreover, these blebs appeared all over the cell membrane, indicating features of apoptosis. Although the role of blebbing under pathological conditions is still debatable, it has been accepted that membrane injury caused during apoptosis may also lead to blebbing [44]. However, morphologically, blebbing could signify a reservoir for an impaired membrane portion, along with its nearby cytosol, and sometimes shows resistance against cell injury [45].

PFTs cause a wide range of ion imbalances that involve Ca^2+^ influx and K^+^ efflux [46,47]. PFT induced toxicity regulates the calcium ion, and variation in its concentrations is critical for cell fate. It is also observed that certain toxins mediate the release of Ca^2+^ from the ER [48,49]. Both the influx and the intracellular release lead to a spike in Ca^2+^ levels. To ascertain this, we performed Fluo-4 AM staining to detect the changes in Ca^2+^ levels. Upon exposure to rPLP2, the levels of calcium rapidly increased within the cells, which can be distinguished by the higher level of fluorescence in comparison to the control cells (Figure 3A,B).

Ca^2+^ influx is also associated with the dysfunction of mitochondria, which occurs as a result of calcium stimulation of the mitochondrial TCA cycle [35]. This can lead to ROS production, ATP depletion, and eventually cell death. Hence, to monitor the effect of rPLP2 on the cells, we used Annexin/PI dual staining. Our results indicate that rPLP2 mediates a late apoptotic/necrotic phenotype that can be deciphered from Annexin/PI dual positivity (Figure 4A). Further downstream analysis indicated a drastic reduction for ΨΔm in rPLP2 treated cells compared to the control cells, which had intact potential (Figure 4C,D).

ROS production is one of the markers for overstimulation of the mitochondrial TCA cycle. They can further permeabilize the mitochondria by positive feedback [50], as well as disrupt the lysosomes [51]. Many studies have determined the role of ROS as a mediator for cell death in the case of PFTs [47,52]. An increase in levels of ROS also disrupts the blood–brain barrier during cerebral malaria. In this study, we could also clearly detect increased levels of ROS in the rPLP2 treated cells (Figure 5A). Importantly, malaria patients have been reported to have lower catalase activity than healthy controls, but a higher SOD activity, thus resulting in the accumulation of H_2_O_2_. Moreover, free heme released from the hemoglobin of ruptured parasite infected red cells may continuously undergo autoxidation, producing superoxide, which dismutates into H_2_O_2_ and is a potential source for subsequent oxidative reactions in endothelial cells [53].

It is well known that increased ROS level leads to DNA damage and decreased energy production [54]. All of these factors finally lead to the death of the target cell. After cellular membrane damage, the release of intercellular contents, including HMGB1, is a cause of inflammation in necrosis [55]. The depletion of ATP occurs due to the over-activation of PARP, causing necrosis. ROS leads to DNA damage, leading to activation of PARP, which further phosphorylates HMGB1 [56]. Upon staining for HMGB1, we found the levels were largely located in the nucleus in control cells (Figure 5C). However, a clear translocation from the nucleus to the cytosol (Figure 5C,D) was evident in the treated cells, indicating necrosis.

Taken together, we hypothesize that the release of PLPs in blood plasma compromises the barrier cell integrity. The addition of purified rPLPs leads to an increase in intracellular Ca^2+^, loss of mitochondrial membrane potential, and cell death. This study indicates the probable role of PLPs in the dysfunction of EC during severe malaria. Furthermore, this work provides an in vitro model for the study of EC dysfunction that can be attributed to extreme malaria.

## 4. Materials and Methods

### 4.1. Cell Culture

Madin-Darby canine kidney (MDCK) cells were procured from the National Centre for Cell Science, Pune, India. The MDCK cell line was maintained in DMEM (Thermo Fischer Scientific, Waltham, MA, USA), supplemented with 10% FBS (Thermo Fischer Scientific, Waltham, MA, USA) and 1% penicillin/streptomycin (Thermo Fischer Scientific, Waltham, MA, USA). Human umbilical vein endothelial cells (HUVECs) were purchased from Himedia (Mumbai, India) and cultured in EGM2 media (Lonza, Basel, Switzerland) supplemented with growth factors (Lonza, Basel, Switzerland).

### 4.2. Recombinant Protein Purification

The purification of rPLP2 [27] and rEtx [29] was carried out as described previously. In general, the codon-optimized gene encoding for rPLP2 was subcloned into pET28a (+), and protein expression in *E. coli* cells was induced with 1 mM of isopropyl-β-D-thiogalactoside (IPTG) (Sigma Aldrich, St. Louis, MO, USA). The histidine tagged protein was purified using Ni-NTA affinity chromatography, and the concentration was determined using the BCA estimation kit (Thermo Fischer Scientific, Waltham, MA, USA).

### 4.3. Cell Viability

MDCK cells were cultured in 96-well plates at a density of 0.2 × 10^5^ cells/well to analyze the effects of purified rPLP2 or rEtx. Serial dilutions of the proteins in incomplete DMEM (iDMEM, FBS-free, antibiotic-free DMEM) were incubated with the cells in triplicates for 1 h at 37 °C. The control wells had an equal volume of media. To analyze the effect of EGTA (Calbiochem, San Diego, CA, USA) and LaCl_3_ (Sigma Aldrich, St. Louis, MO, USA), the plate was incubated with 2 mM of EGTA and 100 μM of LaCl_3_ for 15 min, followed by the addition of 75ng/mL of rEtx. After the incubation for 1 h at 37 °C, the cell viability was measured using MTT (3-(4,5-dimethylthiazol-2-yl), 2,5diphenyltetrazolium bromide) (Himedia, Mumbai, India), and the absorbance was read at A_550_ using a Biorad iMark microplate reader (Biorad, Hercules, CA, USA). Untreated cells were taken as 100% cell survival, and the viability for toxin treated cells was calculated accordingly.

### 4.4. Endotoxin Contamination Assay in Recombinant Protein

The level of endotoxin in the *E. coli* purified recombinant protein was estimated by the Pierce LAL Chromogenic endotoxin quantification kit (88282, Thermo Scientific, Waltham, MA, USA) following the manufacturer’s instructions. Briefly, rEtx, rPLP2, and *E. coli* endotoxin standards were taken (50 µL each), and 50 µL of the LAL reagent was added to each tube, followed by incubation at 37 °C for 10 min. For detection, 100 µL of the chromogenic substrate was added and further incubated at 37 °C for 6 min. The reaction was stopped by the addition of 100 µL of 25% acetic acid, and then absorbance was measured at 405 nm in the Tecan M200 plate reader (Zurich, Switzerland).

### 4.5. RBC Lysis Assay

Five million human erythrocytes were incubated with rPLP2 or rPLP2 denatured (purified from inclusion bodies) in lysis buffer for 1 h at 37 °C, as described previously [21]. The release of hemoglobin into the supernatant was estimated by measuring the absorbance at 405 nm. The hemoglobin released by the lysis of 5 × 10^6^ human RBCs in water was considered as 100% lysis, and the values for protein treated erythrocytes were plotted accordingly.

### 4.6. Live-Cell Video Microscopy

MDCK or HUVEC were plated on glass bottom culture dishes (ibidi, Gräfelfing, Germany) and incubated at 37 °C. For blebbing, the recording was initially carried out for 5 min at 37 °C, followed by the addition of rPLP2. The morphological changes that occurred were observed in real time using a Nikon confocal microscope (Nikon A1R, Nikon Corporation, Tokyo, Japan) equipped with a temperature controlled stage. For calcium imaging, the cells were loaded with 3 µM of Fluo-4 AM (Thermo Fischer Scientific, Waltham, MA, USA) in the dark for 20 min at 37 °C, followed by two washes to remove the excess dye. The cells were then treated with rPLP2 for 30 min and imaged under the microscope at 37 °C. For live-cell microscopy, the cells were loaded with Fluo-4 AM as described. rPLP2 was added to the cells, and the time-lapse video microscopy was carried out for 10 min using a 1.4 numerical aperture lens and analyzed by Nikon ES elements software (Nikon, Nikon Corporation, Tokyo, Japan).

### 4.7. Erythrocyte Calcium Assay

Erythrocytes were loaded with 5 µM of Fluo-4 AM (Thermo Fischer Scientific, Waltham, MA, USA) in the incomplete RPMI (iRPMI without albumax and antibiotics) for 20 min at 37 °C. After dye loading, the erythrocytes were treated with rPLP2 in iRPMI for 20 min at 4 °C for binding, followed by two washes with ice-cold iRPMI. To analyze the effect of calcium chelation and blockage, the erythrocytes were incubated with 2 mM of EGTA and 100 μM of LaCl_3_ at 37 °C for 45 min. The erythrocytes were then quantified using FACS BD Fortessa (Becton & Dickinson, Franklin Lakes, NJ, USA) using Cell Quest software by scoring 100,000 cells per sample. The samples were analyzed using FlowJo software (FlowJo, LLC, Ashland, OR, USA) by determining the proportion of FL-2 positive cells in comparison to the stained untreated erythrocytes. The percentage of Fluo-4 AM positive rPLP2 treated erythrocytes was considered 100% positive, and the other samples were plotted accordingly.

### 4.8. Annexin-PI Staining

MDCK cells were cultured on sterile coverslips (Corning, NY, USA) and incubated at 37 °C overnight. Once the cells reached 70–80% confluency, they were treated with the protein for 1 h at 37 °C. After incubation, the cells were washed with PBS and stained with Annexin V-FITC (Thermo Fischer Scientific, Waltham, MA, USA) and PI (Thermo Fischer Scientific, Waltham, MA, USA) according to the manufacturer’s protocol. Briefly, the cells were incubated with 1X Annexin V-FITC and 2 µg/mL PI for 15 min, followed by imaging at 37 °C.

### 4.9. Mitochondrial Membrane Potential and ROS Detection Assays

The cells were seeded on the glass bottom culture dishes (ibidi, Gräfelfing, Germany) and incubated at 37 °C overnight. Once the desired confluency was reached, the cells were incubated with the protein. After 1 h, the cells were washed and stained with 2 µM of JC-1 (Thermo Fischer Scientific, Waltham, MA, USA) for 15 min, followed by imaging at 37 °C. Similarly, for ROS detection, the cells were incubated with 5 µM of DCFDA (Thermo Fischer Scientific, Waltham, MA, USA) after the treatment with the protein and imaged under the confocal microscope (Nikon A1R, Nikon Corporation, Tokyo, Japan).

### 4.10. HMGB1 Assays

MDCK cells were grown on the sterile coverslips (Corning, NY, USA) and incubated at 37 °C overnight. The cells were treated with the protein for 1 h, and the coverslips were washed and fixed with 2.5% paraffin for 20 min, followed by permeabilization with 0.5% Triton X-100 in PBS for 5 min. HMGB1 (1:500, Thermo Fischer Scientific, Waltham, MA, USA) was used as a primary antibody, followed by the addition of anti-rabbit Alexa 488 or anti-rabbit Alexa 594 (Thermo Fischer Scientific, Waltham, MA, USA) as the secondary antibody. The coverslips were mounted on a glass slide using DAPI antifade (Thermo Fischer Scientific, Waltham, MA, USA) and imaged under the confocal microscope (Nikon A1R, Nikon Corporation, Tokyo, Japan).

### 4.11. Statistical Analysis

The student’s t-test was performed wherever applicable, and *p*-values of <0.05, <0.01, and <0.005 were considered significant (denoted by *, **, and ***), respectively. Results represent the mean ± SD of three independent experiments.

## Supplementary Materials

The following are available online at https://www.mdpi.com/2072-6651/13/1/62/s1, Figure S1: Endotoxin contamination and erythrocyte lysis measurement, Figure S2: Blebbing of cells in response to rPLP2, Figure S3: Biological activity of the recombinant proteins in the presence of EGTA and LaCl_3_, Figure S4: Effect of rPLP2 on the mitochondrial membrane potential of MDCK cells. Video S1: Blebbing of MDCK cells in response to rPLP2. The MDCK cells were treated with rPLP2 and time-lapse video microscopy was performed to detect the change in the membrane architecture, Video S2: Blebbing of HUVEC in the presence of rPLP2. Time-lapse video microscopy was carried out to observe the modifications in membrane architecture upon exposure to rPLP2, Video S3: Increase in calcium levels in response to rPLP2. Fluo-4AM loaded HUVEC were treated with rPLP2 and the increase in the levels of calcium was detected by time-lapse video microscopy.
