# Supplementary material for: Pathogenic Pore Forming Proteins of Plasmodium Triggers the Necrosis of Endothelial Cells Attributed to Malaria Severity
Source: Toxins (Basel). 2021 Jan 15;13(1):62. doi: 10.3390/toxins13010062 (PMC7839052; doi:10.3390/toxins13010062)
Supplement: Supplementary file 1 [file toxins-13-00062-s001.zip › toxins-921023 supplementary materials/toxins-921023 supplementary figures.docx]

Supplementary Materials: Pathogenic Pore Forming Proteins of *Plasmodium* Triggers the Necrosis of Endothelial Cells Attributed to Malaria Severity

Abhishek Shivappagowdar, Swati Garg, Akriti Srivastava, Rahul S. Hada, Lalit C. Garg, Soumya Pati and Shailja Singh


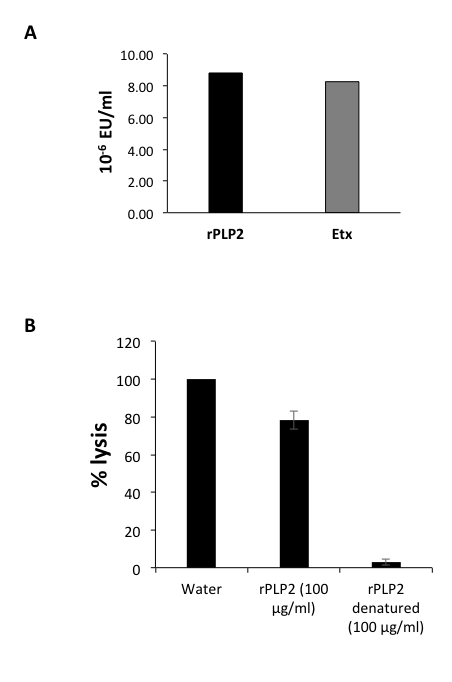


**Figure S1.** Endotoxin contamination and erythrocyte lysis measurement. (**A**) Endotoxin contamination level in rPLP2 and rEtx protein were measured using an Endotoxin Quantitation kit. The graph indicates the endotoxin contamination (EU/mL) in the final treated HUVEC cells. (**B**) Erythrocyte lysis mediated by rPLP2. The lysis of human erythrocytes was analyzed in the presence of active and denatured rPLP2 (purified from inclusion bodies). The graph indicates the percent lysis of human erythrocytes by the proteins as compared with the 100% hypotonic lysis of human erythrocytes in water.


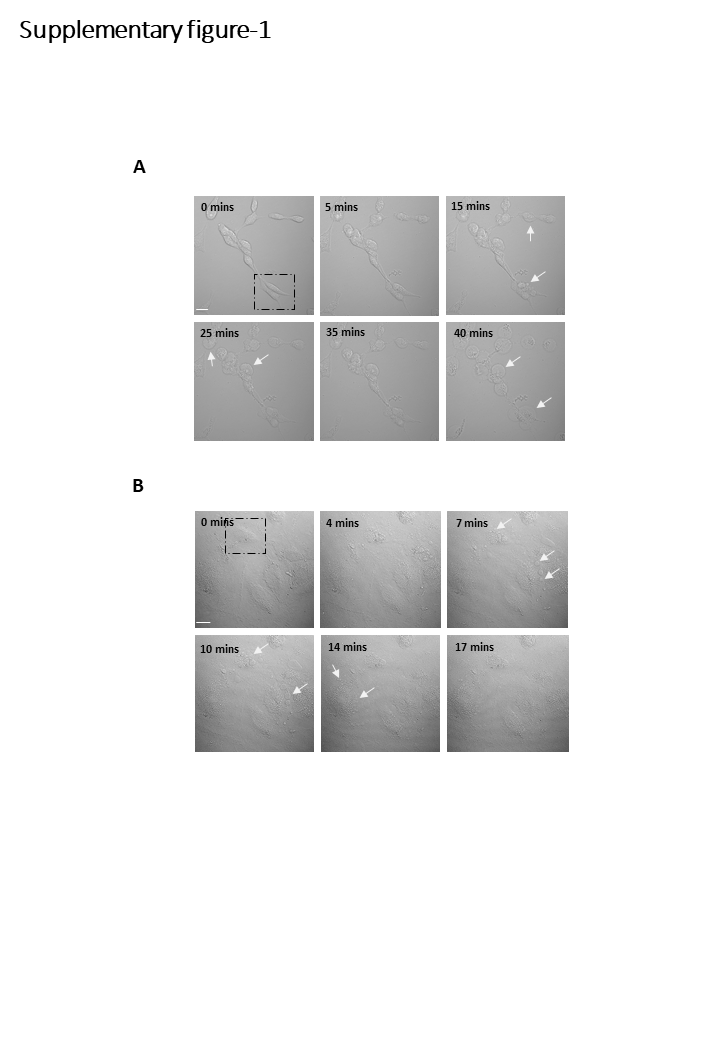


**Figure S2.** Blebbing of cells in response to rPLP2. The MDCK (**A**) and HUVEC (**B**) were treated with rPLP2 (10 µg/mL) and live-cell microscopy was performed to observe the changes in the membrane architecture. The snapshots of the complete frames are shown here. The boxed region indicates the area cropped and shown in Figure 2. The white arrows indicate the formation of blebs. The scale bar represents 10 μm.

**A**

**B**


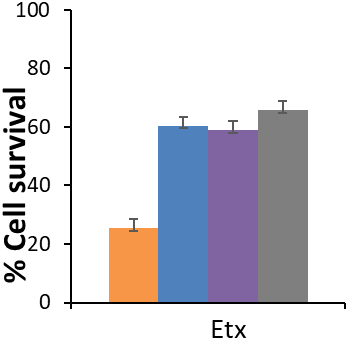


**

*

**

Etx

Etx+EGTA

Etx+LaCl_3_

Etx+ EGTA+LaCl_3_

**Figure S3.** Biological activity of the recombinant proteins in the presence of EGTA and LaCl_3_. (**A**) The erythrocytes were treated with rPLP2 (5 µg/mL) alone at 4 ^°^C followed by treatment with or without EGTA and LaCl_3_. The Fluo-4AM positive erythrocytes were quantified using a flow cytometer. (**B**) MDCK cells were treated with the Etx (75 ng/mL) in the presence or absence of EGTA and LaCl_3_. The viability of the protein treated cells was analyzed by MTT assay. Statistical significance is shown for the indicated groups vs Etx (**P* < 0.05, ***P* < 0.01, *** *P* < 0.005; Student’s t-test). The data represent mean ± SD.


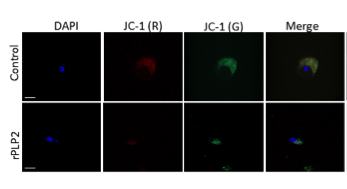


**Figure S4.** Effect of rPLP2 on the mitochondrial membrane potential of MDCK cells. MDCK cells were loaded with JC-1 and treated with rPLP2 (5 µg/mL). The effect of the protein on the mitochondrial depolarization was observed by confocal microscopy. The scale bar represents 10 μm.
